# Possibilities for Groundwater Flow Sensing with Fiber Bragg Grating Sensors

**DOI:** 10.3390/s19071730

**Published:** 2019-04-11

**Authors:** Sandra Drusová, Wiecher Bakx, Adam D. Wexler, Herman L. Offerhaus

**Affiliations:** 1 Wetsus, European Centre of Excellence for Sustainable Water Technology, Oostergoweg 9, 8911 MA Leeuwarden, The Netherlands; wiecher.bakx@arcadis.com (W.B.); adam.wexler@wetsus.nl (A.D.W.); 2 Optical Sciences, University of Twente, Hallenweg 23, 7522 NH Enschede, The Netherlands; h.l.offerhaus@utwente.nl; 3 Arcadis Nederland B.V., Beaulieustraat 22, 6814 DV Arnhem, The Netherlands

**Keywords:** fiber Bragg grating, aquifer simulator, thermal tracer, FBG interrogators, multiplexed temperature sensing

## Abstract

An understanding of groundwater flow near drinking water extraction wells is crucial when it comes to avoiding well clogging and pollution. A promising new approach to groundwater flow monitoring is the deployment of a network of optical fibers with fiber Bragg grating (FBG) sensors. In preparation for a field experiment, a laboratory scale aquifer was constructed to investigate the feasibility of FBG sensors for this application. Multiparameter FBG sensors were able to detect changes in temperature, pressure, and fiber shape with sensitivities influenced by the packaging. The first results showed that, in a simulated environment with a flow velocity of 2.9 m/d, FBG strain effects were more pronounced than initially expected. FBG sensors of a pressure-induced strain implemented in a spatial array could form a multiplexed sensor for the groundwater flow direction and magnitude. Within the scope of this research, key technical specifications of FBG interrogators for groundwater flow sensing were also identified.

## 1. Introduction

The majority of groundwater reserves are stored within porous sedimentary structures and fractured rocks [1]. Groundwater is often extracted from underground aquifers via drilled wells and globally provides almost 50% of all drinking water and 43% of all consumptive use in agriculture [2]. The careful assessment of local hydrogeological conditions is necessary when drilling, installing, and operating a groundwater extraction well. Understanding groundwater flow near wells helps to identify the mechanisms of groundwater recharge and to estimate water resources for extraction. Accurate flow information helps to prevent well clogging, salt water intrusion in coastal areas, or contamination of the drinking water. Natural groundwater velocities typically do not exceed a few meters per day [3]. Therefore, groundwater-related problems are slow to develop and may require months or years to detect. Once identified, the mitigation of the problem can take years and is usually accompanied by high costs [4,5]. This is why all groundwater sources should be monitored and protected.

Groundwater flow velocities are a fundamental input to flow models and are typically measured using in situ methods. Two of the most common methods are discrete flow meters and tracer dilution tests. Regardless of the method chosen, boreholes must be drilled in the area of study and can potentially disrupt the natural flow of groundwater. Flow logging using discrete flow meters placed in a borehole is the current industry standard [6]. These devices use a variety of operating principles including electromagnetic, acoustic, impeller, or heat pulses to measure velocity [7]. The selection of a suitable tool for flow monitoring relies on a priori information about hydrogeological conditions, which is not always available [8]. Tracer tests work by adding a concentrated tracer at a constant rate for a predetermined period of time into a borehole upstream, and flow velocity is determined as a function of a tracer arrival time in downstream boreholes. The velocity can be also determined with a single borehole measurement by monitoring the concentration of a tracer as a function of time due to dilution [9]. Tracer tests are a powerful technique for identifying flow patterns and the hydraulic conductivity of aquifers, as they not only provide velocities but can also identify preferential flow paths in the area [6]. However, especially at low groundwater flow rates, completing tracer tests can be time-consuming.

Heat is becoming more utilized as a tracer for three primary reasons: speed, cost, and environmental friendliness [10]. Measuring the temperature is fast and less costly than measuring the solute concentration. Moreover, it is an environmental tracer—variations in groundwater temperature already occur naturally due to the surface water or fracture inflows [11]. Active heat tracing experiments involve either heating a discrete volume of groundwater or injecting hot/cold water into an aquifer [12,13]. While this approach is generally considered to be environmentally safe, high temperature changes may have an effect on the chemical properties and microbiological stability of aquifers [14]. When relatively large temperature differences are created in the subsurface, the resulting changes in density and viscosity modify the flow regime [15]. The collection of spatial temperature data from the field has, until recently, been a limitation to heat tracing methods. Innovations in optical fiber sensing technology have opened new possibilities for this method by improving spatial and temporal resolutions.

Optical fibers have quickly gained acceptance as temperature sensors due to their high resolution. Their main advantage is their serial multiplexing capability—a single fiber can provide a large number of highly accurate temperature measurements along the entire length, reducing the costs of the deployment. Their small size and durability open up the possibilities for embedding them inside materials. One optical fiber-based technology that is becoming more widely implemented in hydrogeological studies is distributed temperature sensing (DTS). In DTS, the temperature is calculated from the intensity of inelastically backscattered light propagating in an optical fiber [16]. The temperature readings are spatial averages from discrete sections in the fiber. DTS in combination with hot water as a tracer was used to characterize groundwater-surface inflows [17], vertical groundwater flows in boreholes [18,19], and horizontal groundwater flow in an unconsolidated aquifer [20].

**Figure 1:**
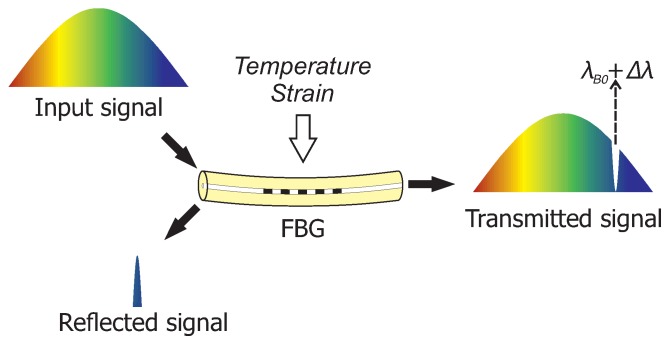
The basic sensing principle of an fiber Bragg grating (FBG) sensor.

Another fiber-optic technology with a potential use for groundwater flow monitoring is fiber Bragg grating (FBG) sensors [21,22]. FBG sensors already have a broad application range in aerospace (load monitoring and shape sensing [23,24,25]), in civil engineering (structural health monitoring [26,27,28]), and in the oil and gas industry (temperature and pressure monitoring [29]). An FBG sensor is a periodic variation of the refractive index within a fiber core which acts like a wavelength-specific mirror. When an FBG sensor is illuminated by a broadband light source, part of the light is reflected when it satisfies the Bragg condition [30]:

(1) $$\lambda_{B}=2\cdot n_{eff}\cdot \Lambda_{G}$$

where $\lambda_{B}$ is a reflected Bragg wavelength, $n_{eff}$ is an effective refractive index for the propagating light, and $\Lambda_{G}$ is a grating period. Changes in the temperature or mechanical strain affect both the refractive index of the fiber and the grating period, causing a shift Δλ in the initial Bragg wavelength $\lambda_{B0}$ (Figure 1). Multiple physical quantities can be measured with FBG sensors when their influence is translated into strain or temperature variations, e.g., pressure, vibrations, and curvature of the fiber [31,32]. FBG sensors provide instantaneous and point-wise measurements.

A choice between FBG and DTS sensing depends on the intended application. DTS fibers are usually standard single-mode or multimode fibers used in the telecom industry, which makes them affordable in large lengths. FBG sensors can be written in standard and specialty optical fibers, and the fiber price increases per number of sensors. Fiber interrogators for collecting the DTS sensing data still remain around five times more expensive than FBG units. Current DTS systems provide continuous measurements along the fiber length with intervals of 12.5 cm, a spatial resolution of 30 cm, an accuracy of 1 ${}^{{}^{\circ}}$C, and a temperature resolution of 0.01 ${}^{{}^{\circ}}$C [33,34,35]. The temperature resolution of an FBG sensor is typically determined by the resolution of the interrogator and the sensitivity of a sensor to temperature changes. The current state-of-the art interrogators provide around 1 pm spectral resolution [36] corresponding to a 0.1 ${}^{{}^{\circ}}$C change in bare fibers. The temperature sensitivity of an FBG sensor can be improved through packaging [37,38,39]. The location of FBG sensors in a fiber can be tailored by the user to a submillimeter spatial resolution. The temperature calibration of both DTS and FBG sensors needs to be performed to acquire the value of temperature sensitivity coefficients for a given packaging.

The groundwater temperature is stable, and variations smaller than 0.1 ${}^{{}^{\circ}}$C are insignificant for heat tracing experiments. Differential temperature measurements are more important than absolute values due to small variations in background groundwater temperature. Active heat tracing experiments require heating the injected water between 10 and 80 ${}^{{}^{\circ}}$C in order to compensate for the heat losses by heat diffusion in the sediment [40]. In order to determine whether a sensor can be used for a particular application, one must consider the following parameters: resolution, dynamic range, accuracy, and stability. Standard FBG sensors already have a sufficient temperature resolution to measure groundwater temperature changes without additional packaging. The required dynamic range is determined by the expected temperature difference between the heat pulse magnitude and aquifer temperature. The measurement accuracy is affected by the temperature calibration process and FBG interrogator characteristics. Generally, a temperature-stabilized FBG interrogator is required for groundwater flow measurements, and the long-term wavelength stability needs to be evaluated under field conditions.

Current flow models are limited by an insufficient spatial sampling of the heterogeneous subsurface. In order to better manage an increased aquifer use, e.g., extraction and thermal energy storage systems, more precise flow sensing techniques need to be deployed. An ideal groundwater flow monitoring system should provide a reliable long-term operation and give real-time warnings for abrupt changes. These conditions could be fulfilled by a distributed network of FBG sensors producing a 3-D map of the groundwater flow field. FBG temperature and strain sensing capabilities were validated during a heat tracing experiment, which was performed in a laboratory scale aquifer. The accuracy and suitability of FBG sensors for groundwater flow monitoring has been evaluated in this paper.

## 2. Materials and Methods

An aquifer simulator (AS) was used in the laboratories of Deltares, Delft, the Netherlands to investigate the feasibility of FBG sensors for groundwater flow monitoring applications. This section describes the experimental setup, the calculation of temperature from FBG data, and the temperature calibration process.

### 2.1. Aquifer Simulator and FBG Fibers

Aquifer simulators are commonly used to validate groundwater sensors under realistic conditions on a small scale. An aquifer simulator consists of a watertight container filled with a porous sediment saturated with water. Controlled groundwater flow conditions have previously been used to study the migration of a contaminant plume [41,42] and the temperature distribution in a dike [43] or to evaluate the accuracy of flowmeters measuring the horizontal groundwater velocity [8]. The AS used in this study was constructed from a metal frame with glass walls measuring 2 × 1 × 1 m (length × width × height) which was filled with sand of 0.1–0.25 mm grain sizes, a porosity of 0.41, and a hydraulic conductivity of 9 m/d. The sand layer was deposited by hand in order to reduce the variation in sediment structure and was subsequently sealed with a 4-cm-thick clay layer, forcing the water to flow through the sand rather than over the top. The base was filled with a coarser sand and covered with a plastic sheet.

A water flow through the sand was created by a hydraulic head gradient between the inflow and outflow reservoirs. Water in the inflow reservoir was pumped from a feed reservoir and had an overflow at a controlled height, keeping the inflow water level stable. An even distribution of the flow into the sand both at the inflow and outflow sides of the system was created using six vertically placed perforated tubes with vertical slots of a 0.5-mm width. Hydraulic head differences were checked by two piezometer tubes near inflow and outflow inside the AS. The flow in the simulator was expected to be laminar with some local variations due to differences in the way the sediment settled and due to an obstruction by the frame. The mass discharge $\dot{m}$ of water with density ρ through an AS with a cross-sectional area *A* was measured regularly to determine the flow velocities *v*:

(2) $$v=\frac{\dot{m}}{\rho \cdot A}$$

**Figure 2:**
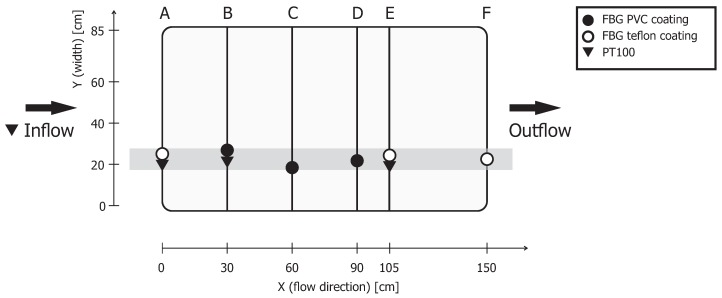
The position of the FBG fibers and PT100 sensors on the PVC frame: The top view. The data from the highlighted section are displayed in the results.

FBG fibers in the sediment were attached to a frame of polyvinyl chloride (PVC) tubes to ensure the placement of the sensor strings with respect to each other (Figure 2). The FBG interrogator used for the experiment was capable of simultaneously interrogating 8 sensors. For this reason, all FBG fibers had 8 FBG sensors each, with Bragg wavelengths of 1518 nm, 1527 nm, 1536 nm, 1545 nm, 1554 nm, 1563 nm, 1572 nm, and 1581 nm. The FBG sensors were inscribed in a standard single-mode Corning SMF28 fiber with an acrylate coating using an ultraviolet femtosecond laser (Loptek GmbH & Co., KG, Berlin, Germany). Two types of FBG fibers were used in the AS: 3 fibers (B, C, and D) had a sensor spacing of 15 cm and a PVC buffer of 3 mm in diameter, and 3 fibers (A, E, and F) had a 10-cm spacing of the sensors and a 1-mm-thick teflon tube of 3 mm diameter in the sensor section. Nine resistive platinum PT100 temperature sensors were placed in the AS as an additional temperature reference plus one PT100 sensor in the inflow reservoir (FS400P, Conrad). The data from the PT100 sensors was acquired by an Ecograph RSG30 (Endress + Hauser, Reinach, Switzerland).

**Figure 3:**
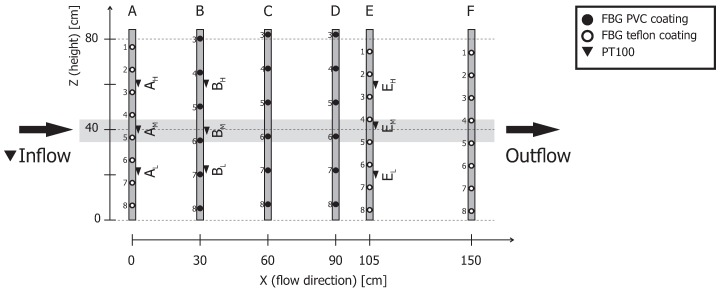
The position of all FBG and PT100 sensors in the aquifer simulator (AS): The side view.

The distribution of the FBG fibers in the X (flow) direction was almost equal, with a spacing of 30 cm (see Figure 2). The only exception was row E. This row was shifted 15 cm towards the inflow to create more space between rows E and F for the experiments with a fiber placement in sand as a preparation for future field studies. One of the experimental tools for the placement of the fibers was a hollow push rod with a cone-shaped driving point. The nonuniform distribution of FBG sensors in the Z direction (height) was a result of the fiber mounting procedure (see Figure 3). The fibers were taped on the two ends to the PVC frame to allow a certain degree of freedom to prevent fiber damage with the heavy sand load. The fibers slipped up to 5 cm compared to the initial location during the attachment and stretching (all selected FBG sensors for the data were within 5 cm distance from each other). The taped fibers also adapted their shape as the container was filled with sand; this was why the displayed results were from a 10-cm interval in the middle of the setup, as it was not possible to guarantee an equal distribution in the aquifer simulator.

### 2.2. FBG Data Acquisition and Temperature Calculation

The data from the FBG sensors was collected using an FBG interrogator (Gator, Technobis, Alkmaar, The Netherlands). The interrogation was performed by a photonic integrated circuit with an arrayed waveguide grating. The interrogator emited broadband light in the range of 1516–1584 nm into the fiber. The reflected signal was directed by an optical circulator to an arrayed waveguide grating serving as a demultiplexer for up to 8 Bragg wavelengths. A proprietary center-of-gravity algorithm calculated the peak Bragg wavelengths from the measured photodiode current. A 1 × 16 fiber optic switch (eol 1 × 16, LASER COMPONENTS GmbH, Olching, Germany) was used to extend the number of interrogated fibers in the system. Both the switch and the interrogator were supervised by a microcontroller (Raspberry Pi Model 3B, Raspberry Pi Foundation, Cambridge, UK) using a Python serial library.

A sampling period of 10 s was chosen to yield enough sampling points to capture the heat processes in the sediment in great detail. The interrogator continuously sent the reflected wavelengths information via USB at a frequency of 1 kHz, overwriting previous data within the device buffer. The 1-kHz sampling interval was too high for the desired application, but this factory setting could not be changed. Fifty datasets were acquired every 10 s and averaged to increase the accuracy.

**Figure 4:**
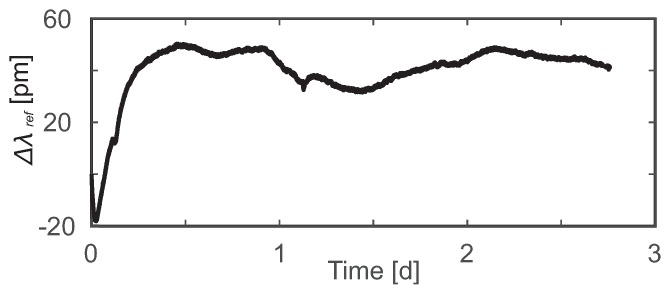
The long-term wavelength drift of the FBG interrogator at 1550 nm as measured with a temperature-controlled FBG sensor.

The groundwater temperature was very stable and rarely fluctuated 0.1 ${}^{{}^{\circ}}$C in an hour; thus, a long-term stability of the interrogator light source was required for the heat-tracing experiments. According to the published interrogator specifications, the wavelength stability was 5 pm in a steady-state environment [44]. However, a measurement with the FBG fibers in a stable laboratory environment showed a wavelength variation up to 20 pm, which translated to a drift of 2 ${}^{{}^{\circ}}$C. The measured drift did not originate in the fiber but was rather due to the thermal instabilities affecting the interrogator photonic integrated circuits. At the time of this study, the interrogator had no built-in wavelength referencing system so the manufacturer provided an additional external reference. This external reference was an FBG sensor with a Bragg wavelength of 1550 nm surrounded by thermoelectric elements controlled by a thermoelectric cooling (TEC) controller (ITC4005, Thorlabs, Newton, MA, USA). With this FBG reference sensor embedded, immobilized, and temperature-stabilized, the reflected wavelength could be used to quantify the drift of the system at 1550 nm directly (see Figure 4). The drift was approximately 70 pm during the initial 12-h operational period and, afterwards, fluctuated in the range of 20 pm. Thus, the interrogator could be used for the heat-tracing experiments only in combination with the external reference sensor since a drift correction needed to be performed.

Since the FBG sensors had Bragg wavelengths different from the reference, the wavelength-dependent drift could not be directly subtracted from the measured data. An additional correction or gain factor, *G*, needed to be determined, which scaled the measured FBG wavelengths to match the reference:

(3) $$G=\frac{\lambda_{B}}{\lambda_{ref}}$$

where $\lambda_{B}$ is the Bragg wavelength from an FBG sensor and $\lambda_{ref}$ is the Bragg wavelength of the reference FBG sensor. The wavelength dependency of *G* was investigated using the AS under the following conditions: the AS was off, the temperature inside was stable, and there were no factors that could introduce strain effects in the sediment. In this case, the data collected from the FBG sensors directly represented the system drift at those corresponding Bragg wavelengths. The experiment showed that G=G(λ) is linear; therefore, the gain value for any FBG sensor can be simply calculated as a constant:

(4) $$G=\frac{\lambda_{B0}}{\lambda_{ref0}}$$

where $\lambda_{B0}$ is the initial Bragg wavelength of an FBG sensor and $\lambda_{ref0}$ is the initial Bragg wavelength of the reference sensor. The Bragg wavelength shift Δλ with respect to a change of the external temperature ΔT and the strain Δϵ is described by the following equation [30], with the reference term subtracted:

(5) $$\frac{\Delta \lambda }{\lambda_{B0}}-G\frac{\Delta \lambda_{ref}}{\lambda_{ref0}}=\left(1-p\right)\Delta \epsilon +\left(\alpha_{e}+\alpha_{n}\right)\Delta T$$

where *p* is a strain-optic coefficient, $\alpha_{e}$ is a thermal expansion coefficient (describes changes in the grating length due to temperature), and $\alpha_{n}$ is a thermo-optic coefficient (describes changes in effective refractive index due to temperature). If the fibers are firmly held in the sediment, then the strain effects can be neglected. Relative temperature changes in the AS can be determined from Equation (5) as:

(6) $$\Delta T=\frac{1}{\alpha_{e}+\alpha_{n}}\cdot \left(\frac{\Delta \lambda }{\lambda_{B0}}-G\frac{\Delta \lambda_{ref}}{\lambda_{ref0}}\right)$$

### 2.3. Thermal Calibration of FBG Sensors

The sum of the coefficients $\alpha_{e}+\alpha_{n}$ was obtained experimentally during additional calibration measurements. FBG fibers with two different coatings (in rows A and B) were placed in a calibration water bath with a controlled temperature, together with a reference PT100 sensor (TSP01, Thorlabs, Newton, MA, USA). The water in the bath was heated to +25 ${}^{{}^{\circ}}$C corresponding to the temperature of the hot inflow water entering the AS in the heat-tracing experiment.

The data from the FBG sensors was collected using a different interrogator with a built-in wavelength reference for a more precise calibration (Hyperion si155, Micron optics, Atlanta, GA, USA). The interrogator had a swept laser in the range of 1500–1600 nm scanning the entire spectrum with a 1-kHz frequency. A full optical spectrum was digitized, and the peak wavelengths were calculated with a centre-of-gravity algorithm. With this technology, the number of interrogated FBG sensors is limited by the desired dynamic range. The device has an internal wavelength referencing system consisting of a Fabry–Perot cavity and a gas cell [45]. The measured drift for this interrogator was less than 1 pm.

The temperature sensitivity coefficient α can be calculated from Equation (6), while neglecting the strain and reference term, as

(7) $$\alpha =\alpha_{e}+\alpha_{n}=\frac{\Delta \lambda }{\lambda_{B0}\Delta T}$$

**Figure 5:**
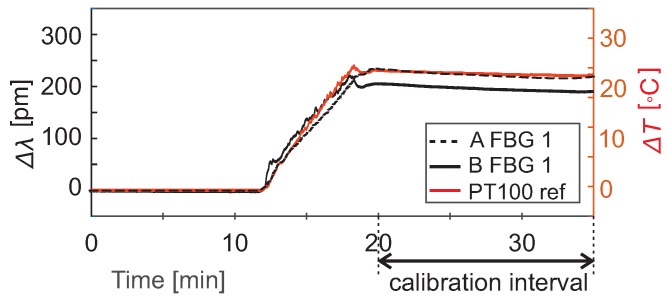
An example of the calibration curves for two FBG sensors: Each sensor is inscribed in a fiber with a different coating. The sensor A FBG 1 has a teflon coating; the sensor B FBG 1 has a PVC coating.

The α values for each coating in Table 1 are an average of 8 FBG sensors within each fiber from a 15-min calibration interval when the water temperature was held stable (Figure 5). The accuracy of the PT100 calibration sensor determined the confidence bounds of the α values. In the interval of 20–45 ${}^{{}^{\circ}}$C, the accuracy of the differential temperature measurement with the reference sensor was ±0.8 ${}^{{}^{\circ}}$C. The temperature sensitivity of the FBG sensors, i.e., the wavelength shift per 1 ${}^{{}^{\circ}}$C, could be calculated from Equation (7). The values for a 1550-nm FBG sensor are presented in Table 1. The temperature resolution of the FBG sensors depends on the resolution of the spectrum analyzer in the interrogator, in this case, 1 pm, and is calculated from the sensitivity *S* as $1\;\left(\mathrm{pm}\right)/S\;\left(\mathrm{pm}{/}^{{}^{\circ}}C\right)$.

The confidence interval for α can be used to calculate the accuracy of the differential temperature measurements with the FBG sensors in the AS. If α changes by Δα, ΔT will change as

(8) $$\Delta T^{\prime }=\frac{\Delta \lambda }{\lambda_{B0}\left(\alpha +\Delta \alpha \right)}$$

The accuracy can, thus, be calculated in comparison to the original ΔT as

(9) $$\frac{\Delta T^{\prime }}{\Delta T}=\frac{\alpha }{\alpha +\Delta \alpha }$$

A varied temperature sensitivity is caused by differences in the thermal expansion properties of the coating material. The thermal expansion coefficient of teflon is slightly larger than that of PVC, $\left(112-135\right)\times 10^{-6}$ (1/${}^{{}^{\circ}}$C) for teflon and $\left(54-110\right)\times 10^{-6}$ (1/${}^{{}^{\circ}}$C) for PVC.

### 2.4. Heat-Tracing Experiments

FBG technology has already been successfully deployed in subsurface environments, but not yet for the purpose of monitoring groundwater flow. One way to achieve this would be to use an array of FBG sensors for localized temperature measurements in heat-tracing experiments. Therefore, a heat-tracing experiment was performed in an idealized version of an aquifer. FBG fibers with two different thermal sensitivities were buried in the AS with a constant flow velocity of 2.9 m/d and varying inflow conditions:

- *Hot inflow.* Time t = 0 h.
  Hot water (48 ${}^{{}^{\circ}}$C) from a feed reservoir was pumped into the inflow reservoir where it slowly mixed with the room temperature (19 ${}^{{}^{\circ}}$C) water before flowing through the inflow pipes and into the AS.
- *Cold inflow.* Time t = 5.5 h.
  In this stage, the feed reservoir was filled with cold tap water (19 ${}^{{}^{\circ}}$C) and pumped into the inflow reservoir.
- *No inflow.* Time t = 7.5 h.
  The feed pump to the inflow reservoir was stopped; both the inflow and outflow systems were closed.

Prior to making measurements, the inflow system was open for two hours to allow the flow to reach a steady-state condition. The flow velocity was chosen to correspond to that of the natural groundwater flow, and we assumed that, for this low velocity, the strain effects would be negligible compared to the temperature effects. Surprisingly, some strain effects were observed and will be discussed below. The temperature and strain cross-sensitivity of the FBG sensors was separated by a comparison with the PT100 sensors.

The wavelength shifts measured by the FBG sensors were translated into temperature, and the results are displayed as

- *1-D temperature profiles*—the time development of a temperature from all sensors along a line in the flow direction (highlighted in Figure 3). A comparison between the PT100 and FBG sensors also allowed the identification of the strain effects.
- *2-D temperature maps*—the time development of a temperature from all sensors along a vertical slice through the AS (all sensors in Figure 3).

## 3. Results and Discussion

### 3.1. Temperature Effects

**Figure 6:**
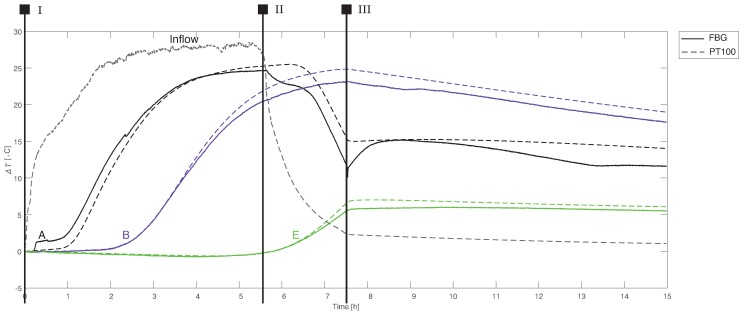
A comparison of the FBG data translated into temperatures with the PT100 temperature profiles: The displayed data are from the sensors highlighted in Figure 3. Identical colors indicate sensors in the same row on the frame (the same distance from the inflow system). Experimental stages: I—hot inflow, II—cold inflow, and III—no inflow.

The majority of FBG signals consists of temperature contributions. Generally, temperature curves from both type of sensors, FBG and PT100, have similar shapes (see Figure 6). The differences are within the accuracy range of the sensors (Table 2). Moreover, the compared sensors were not at identical locations and had different physical dimensions. The PT100 sensors, for example, provided a temperature spatially averaged over 6 cm. Heat transport by convection was a dominant process in stages I and II. Thermal diffusion took place not only in the pore fluid but also in the sand particles. As a result, the advancing thermal tracer front was not a sharp boundary. Diffusion was dominant in stage III; heat transport by diffusion was much slower than convection in this case. A heat exchange between the inflow water, the sand particles, and the AS walls was apparent from the gradual decrease in the maximal temperature as water progressed through the AS.

### 3.2. Thermal Plume Propagation

**Figure 7:**
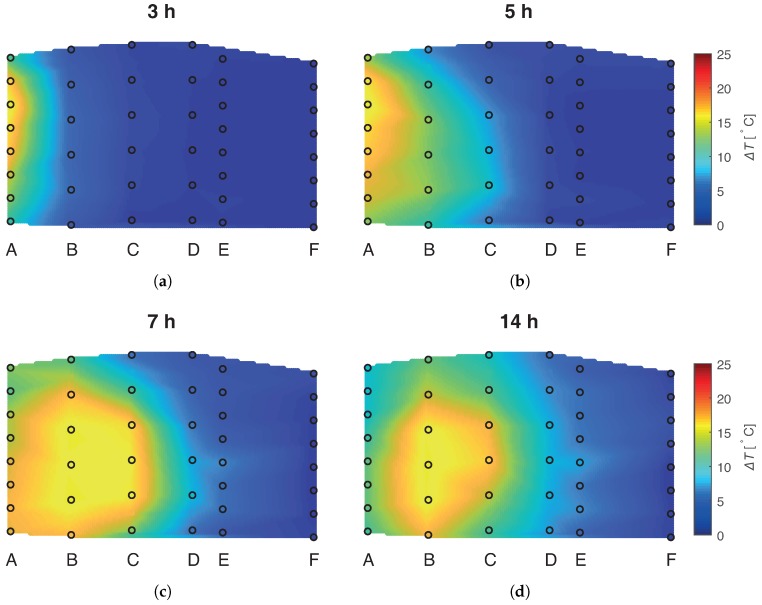
The time evolution of the thermal plume in the AS at four different moments: (**a**) t = 3 h; (**b**) t = 5 h; (**c**) t = 7 h; (**d**) t = 14 h from the start of the experiment. The black circles indicate the calculated temperature from the FBG sensors. The displayed temperature map was generated by a linear interpolation from the FBG data.

The temperature of the inflow water was altered in the experiment, and the FBG sensors were deployed to visualize the propagation of a thermal plume across the AS (see the plume shape in Figure 7). A majority of the strain effects were a fast perturbation in a slow temperature dynamics; therefore, it was possible to choose FBG data not affected by the strain to visualize the thermal plume propagation.

The thermal plume developed asymmetrically over time in the vertical direction and exhibited a quasi-parabolic profile consistent with laminar flow. Initially, the hot plume was centered in the upper section of the AS due to buoyancy effects (see Figure 7a). The hot plume propagated downwards during stages I and II. The heat propagation direction did not coincide with the direction of the flow, possibly due to the asymmetric thermal properties of the upper and lower sediment boundaries. The top of the AS had wet saturated clay as a sealant, and the base was filled with a coarse sand covered with a plastic sheet. The buoyancy effects were expected to prevail during the entire experiment, but this was not the case. This unexpected heat propagation in the sand was captured by the FBG sensors with a high resolution of 0.09 ${}^{{}^{\circ}}$C.

Predicting the propagation of a thermal plume was not a trivial task even in a designed subsurface environment with a quasi-uniform particle distribution and laminar flow. In our case, the heat transport was affected by boundary conditions with different thermal conductivities. The same situation occurred during the heat-tracing tests in the field. A heterogeneity of the subsurface complicated the interpretation of heat-tracing results, as heat does not always propagate in the same direction as the flow. An array of temperature sensors could, then, help to visualize the heat transport in porous sediment structures. The FBG sensors could be used in the heat-tracing tests if they were combined with a certain packaging to eliminate the strain effects (e.g., rigid metal tubing). However, in terms of the cost and resolution, DTS was still a more suitable option for temperature sensing in the subsurface. The use of active heat-tracing methods had been successfully applied to the characterization of groundwater flows in diverse geological formations [12,46]. However, due to the need for a constant power input to run the heating equipment, such an approach is not feasible for long-term flow monitoring. In this regard, FBG flow sensors present an interesting alternative as they also exhibit a sensitivity to strain which would not require power for heating.

### 3.3. Strain Effects

**Figure 8:**
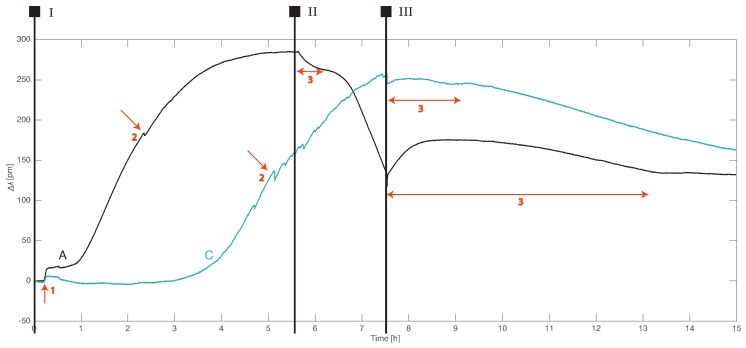
The wavelength shifts of the FBG sensors in rows A and C: The red arrows indicate strain events resulting from (1) setup adjustments, (2) local flow, or (3) global flow changes. The Roman numerals demarcate the different experimental stages consistent with Figure 6.

Prior to this study, it was assumed that the wavelength shifts from FBG sensors in an AS would be caused exclusively by temperature changes. However, as these experiments showed, this was not valid for all FBG sensors. In the case of the FBG sensors in rows A and C, the strain effects were non-negligible (Figure 8). Possible explanations for the observed strain events could be

1. Setup adjustments, e.g., inflow connection check
2. Local flow changes, e.g., preferential flow paths around the sensors or settling of the sediment
3. Global flow changes, e.g., hydraulic head changes

The step strain response at t = 0.2 h (arrow 1) is common for the FBG sensors from rows A and C. It is the result of physical adjustments made to the inflow system. The connection between the inflow reservoir and the inflow tubes is made of several plastic hoses. At the start of the experiment, these connections were checked and tightened. Such manipulations could result in either a physical strain due to the small movements of parts in the AS or changes in the hydraulic conveyance efficiency of the system.

The FGB sensors in rows A and C had the largest strain response at t = 7.5 h (stage III) when the flow in the AS stops—it was a fast step response followed by a slow relaxation with amplitudes up to 50 pm. In the case of fiber A, the slow relaxation lasted for 6.5 h. It is likely that those FBG sensors measured the local pressure (hydraulic head) changes. The strain effect at the start of stage II was caused by a small hydraulic head change when the feed reservoir was drained from hot water. The hydraulic head differential in the AS at the start of stage III was 22 cm and decreased slowly to zero after many hours. Hydraulic head changes near drinking water wells can start from a few tens of cm and reach to several meters depending on how far from the well one measures and the extraction rate. Hence, the strain effects measured by FBG sensors near groundwater extraction wells are expected to be even larger than those found in this study.

FBG sensors are sensitive to pressure if they are translated to axial strain through suitable packaging. The most common design of an FBG pressure sensor uses a perforated metal cylinder to surround the grating which is embedded in an elastic polymer [47]. When both fiber ends are fixed, an increasing pressure compresses the fiber and decreases the grating period, thus causing a negative wavelength shift. FBG sensors in the AS, attached to the PVC frame, are similar to pressure sensors with unequal sensitivities. The FBG sensors in row A were more sensitive than the sensors in row C, while the sensors in the other rows showed no such response. The pressure sensitivity was presumably linked to the amount of pre-strain in the fiber. When the flow in the AS suddenly stopped, the pressure also suddenly increased, causing a drop in the Bragg wavelength (as can be observed in Figure 8). The slow relaxation response (arrows labeled 3) could describe the pressure equalization or elastic response of the fiber material.

Apart from global pressure changes throughout the system, the FBG sensors from rows A and C exhibited abrupt changes with short relaxation times that were localized to one or a few sensors (arrows labeled 2). The amplitude was typically less than 5 pm. These responses could be associated with changes in the local flow due to the nonuniformity of the sand distribution around the fibers or possibly the settling effects. The latter would cause changes in the local radius of curvature as the fiber adapts its shape to the change in sediment distribution. The authors intend to further investigate the sources of the strain in FBG sensors buried in aquifers.

The fact that strain effects are visible for flow velocities as low as 2.9 m/d indicates that pressure–strain-sensitized FBG sensors could be used near drinking water wells with higher flow velocities. Since pressure differences are the driving force for the flow, pressure-induced strains in FBG sensors hold information directly related to groundwater flow. The pressure in the subsurface is not uniform due to a variability in the sediment distribution, and this is where distributed pressure sensors can provide more insight than simple water table measurements. When connected into a network of point sensors, FBG sensors can further offer a possibility to estimate flow magnitude and direction.

## 4. Conclusions

An idealized lab-scale aquifer simulator was constructed to evaluate the suitability of FBG sensors for groundwater flow sensing. The heat-tracing experiment demonstrated that FBG sensors can measure the relative temperature with an accuracy of 0.85 ${}^{{}^{\circ}}$C for the differential measurements. The FBG point sensors used in these experiments provided localized temperature data with a spatial distribution of 10 and 15 cm. Since smooth temperature changes were expected during the heat-tracing experiment, the spatial FBG dataset was linearly interpolated. As a result, the thermal plume propagation in the aquifer could be visualized. An FBG system, as used for these experiments, is capable of a multiple-sensor interrogation with the possibility of an expansion into a larger distributed sensing network.

With these FBG sensors, it is not possible to decouple wavelength shifts caused by changes in the ambient temperature from the fiber strain. If the application requires only the temperature information in an unconsolidated subsurface environment, the FBG sensors should be packaged to remain strain-free and to increase their temperature sensitivity. A different packaging can increase both the temperature and strain sensitivity and/or to decouple both effects. As shown in the results, the strain effects caused by hydraulic head changes are the most significant strain contributions to measure the FBG signal. These effects will be examined and quantified in our future research towards a pressure-based groundwater flow sensor.

A suitable FBG interrogator for groundwater flow monitoring needs to have a low noise level for maximum temperature and strain sensitivity. Currently available interrogators with noise levels under 1 pm are already sufficient for this application. Furthermore, the long-term wavelength stability of a light source in the interrogator needs to be guaranteed. This is only possible when the interrogator is combined with a wavelength reference.

As a first step, the results demonstrated that FBG sensors can be used as temperature sensors in the subsurface environment, but in this implementation, they did not present an advantage over the existing fiber sensing technologies. However, multiplexed FBG pressure or strain sensors have a potential in groundwater flow monitoring. This research will be followed by a field validation and derivation of groundwater flow vectors.

## Figures and Tables

**Table 1:** The results of the thermal calibration: The thermal sensitivities were calculated for two types of FBG coating (packaging) material used in the AS.

FBG Coating	α±Δα (1/${}^{{}^{\circ}}$C)	Sensitivity at 1550 nm (pm/${}^{{}^{\circ}}$C)	T Resolution (${}^{{}^{\circ}}$C)
Teflon	(7.43 ± 0.26) × 10${}^{-6}$	11.51	0.09
PVC	(6.91 ± 0.24) × 10${}^{-6}$	10.71	0.09

**Table 2:** A comparison of the temperature accuracy of the FBG and PT100 sensors used in the heat-tracing experiment.

FBG Coating	ΔT Accuracy (%)	ΔT = 25 ${}^{{}^{\circ}}$C Accuracy (${}^{{}^{\circ}}$C)
Teflon	±3.38	±0.85
PVC	±3.36	±0.84
PT100		±1.1

## Abbreviations

The following abbreviations are used in this manuscript:

FBG	Fiber Bragg grating
DTS	Distributed temperature sensing
AS	Aquifer simulator
TEC	thermoelectric cooling
PVC	polyvinyl chloride
